# Glioblastoma-Initiating Cells: Relationship with Neural Stem Cells and the Micro-Environment

**DOI:** 10.3390/cancers5031049

**Published:** 2013-08-14

**Authors:** Nicolas Goffart, Jérôme Kroonen, Bernard Rogister

**Affiliations:** 1 Laboratory of Developmental Neurobiology, GIGA-Neurosciences Research Center, University of Liège, Liège 4000, Belgium; E-Mail: ngoffart@student.ulg.ac.be; 2 Human Genetics, CHU and University of Liège, Liège 4000, Belgium; E-Mail: Jerome.kroonen@chu.ulg.ac.be; 3 The T&P Bohnenn Laboratory for Neuro-Oncology, Department of Neurology and Neurosurgery, UMC Utrecht, Utrecht 3556, The Netherlands; E-Mail: J.B.G.Kroonen-2@umcutrecht.nl; 4 Department of Neurology, CHU and University of Liège, Liège 4000, Belgium; 5 GIGA-Development, Stem Cells and Regenerative Medicine, University of Liège, Liège 4000, Belgium

**Keywords:** glioblastoma, subventricular zone, cancer stem cells, neural stem cells, micro-environment

## Abstract

Glioblastoma multiforme (GBM, WHO grade IV) is the most common and lethal subtype of primary brain tumor with a median overall survival of 15 months from the time of diagnosis. The presence in GBM of a cancer population displaying neural stem cell (NSC) properties as well as tumor-initiating abilities and resistance to current therapies suggests that these glioblastoma-initiating cells (GICs) play a central role in tumor development and are closely related to NSCs. However, it is nowadays still unclear whether GICs derive from NSCs, neural progenitor cells or differentiated cells such as astrocytes or oligodendrocytes. On the other hand, NSCs are located in specific regions of the adult brain called neurogenic niches that have been shown to control critical stem cell properties, to nourish NSCs and to support their self-renewal. This “seed-and-soil” relationship has also been adapted to cancer stem cell research as GICs also require a specific micro-environment to maintain their “stem cell” properties. In this review, we will discuss the controversies surrounding the origin and the identification of GBM stem cells and highlight the micro-environment impact on their biology.

## 1. Introduction

Malignant gliomas represent some of the greatest challenges in the management of cancer patients worldwide. Primary brain tumors are indeed considered amongst the most refractory malignancies and their most aggressive form, glioblastoma multiform (GBM, WHO grade IV), is also the most common and lethal subtype [1]. Although notable recent achievements have been made in oncology, using state-of-the-art neuroimaging techniques for surgical resections along with multimodal radio- and chemotherapy, the patients’ median survival hardly reaches 15 months from the time of diagnosis [2,3]. This catastrophic survival rate mainly is the consequence of systematic relapses which reflect the failure of the current therapeutic strategies.

Over the last decade, a large number of different treatments were tested but displayed very limited efficacy. One of the most difficult problems in GBM multimodal therapy is to target the largest number of tumor cells. In this context, surgery often is ineffective given the invasive nature of the tumor, making the entire surgical resection of the tumor mass quite impossible without causing harm to the healthy brain. Moreover, particular regions of the brain are hardly amenable to surgical intervention (basal ganglia, brain stem) which makes the prognosis of the disease even worse. On the other hand, chemotherapeutic strategies are associated with several limitations as well. Various factors such as the size of the molecule, the lipophilicity of the drug, the presence of active efflux pumps and the integrity of the blood-brain barrier influence the access of the drug to the brain parenchyma and the tumor itself [4]. Recent studies have indeed demonstrated that the most forceful agents in glioma therapy achieve relatively low concentrations in the tumor surroundings due to the inability of the drug to cross the blood-brain barrier [5,6]. Finally, recent integrated genomic analysis shed the light on the tumor inter- and intra-heterogeneity. Indeed, The Cancer Genome Atlas (TCGA) classifies GBM based on PDGFRA, IDH1, EGFR and NF1 abnormalities in classical, mesenchymal, pro-neural and neural subtypes [7]. Moreover, all of these subtypes could be found in distinct areas of a single tumor as well [8]. The lack of treatment efficacy could therefore be found in this complex intra- and/or inter-tumor genetic heterogeneity.

For years, parallelisms have been made between stem cell biology and oncology, notably because of the growing evidence that genes with important roles in stem cell biology also play a role in cancer. As a result, the concept of a cancer stem cell population (CSCs) was hypothesized; concept in which a relatively small percentage of cells would share characteristics with normal stem cells and display features including maintained proliferation, self-renewal and differentiation abilities. Nowadays, the existence of such fraction of cells, referred to as cancer stem cells or tumor-initiating cells has been described in many tumors [9] including brain cancers [10,11,12] and raised a new hope in order to understand why glioblastomas so systematically relapse. Further down the road, glioblastoma stem cells, or initiating cells (GICs), were notably shown to be involved in experimental tumorigenesis, tumor maintenance and therapeutic resistance [13,14,15,16]. Moreover, this sub-population of cells with tumor-initiating abilities also display neural stem cell (NSC) properties which suggests that NSCs could play a major role in tumor development and sheds the light on the kinship between GICs and NSCs [12]. However, the basic nature of GICs is nowadays still unclear, whether they derive from NSCs, neural progenitor cells or differentiated cells such as astrocytes or oligodendrocytes. On the other hand, NSCs are located in specific regions of the brain called neurogenic niches which retain the ability to produce neurons and glia throughout life, functioning as a source of stem cells and progenitors in adults [17,18]. Those niches are essential to control critical stem cell properties, to feed the NSCs and to support their self-renewal abilities. This “seed-and-soil” relationship has also been adapted to GBM stem cell research, as GICs also seem to require specific interactions with the micro-environment in order to maintain their stem-like properties and their ability to drive tumor growth [19]. Prospective identification and targeting of GICs is thus mandatory in order to fully understand their own biology, to prevent GBM relapses and to develop new powerful therapeutic strategies. In this review, we will debate over the controversies surrounding the origin and the identification of GICs and discuss the impact of the micro-environment on the biology of GICs.

## 2. GBM Origin(s)

**Figure 1:**
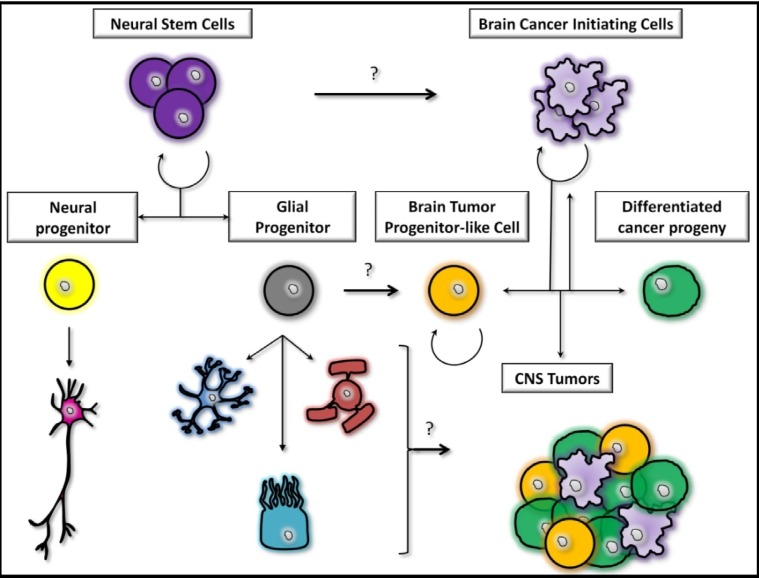
Relationship between NSCs’ differentiation and cancer initiation. NSCs (purlple) are able to differentiate in glial (grey) and neural progenitors (yellow). Neural progenitors give rise to neurons (pink) whereas glial progenitors are committed to oligodendrocytes (red), ependymal cells (light blue) and astrocytes (dark blue). Brain cancer formation could arise from the transformation of NSCs into GICs (Brain Cancer Initiating Cells, light purple) which in turn give rise to a more differentiated cancer cell population (green). In the same line, glial progenitors could induce tumor development after malignant transformation of normal progenitor cells (Brain Tumor Progenitor-like Cells, orange). Traditionally, mature cells in the brain such as neurons, oligodendrocytes, ependymal cells and astrocytes are also seen as potential candidates involved in brain cancer tumorigenesis.

The cells responsible for the onset of malignant gliomas have been source of dissension for many years and are still under intense investigation, whether they could be astrocytes, glial precursors, or stem cells (Figure 1) [20]. In a manner consistent with the stem cell theory, growing evidences aim to demonstrate that only a limited amount of cells, exhibiting stem cell-like properties in the primary tumor, are able to trigger cancer initiation [20,21]. On the other hand, periventricular adult NSCs express high levels of glial fibrillary acidic protein (GFAP) which raised exciting questions on whether or not astrocytes could be involved in GBM initiation. Following those observations, two major hypotheses have been put forward: the astrocytes dedifferentiation theory and the glioblastoma stem cell theory.

### 2.1. The Dedifferentiation Theory

In this hypothetical view, tumorigenesis is regarded as a multi-step process accompanied with genetic alterations which lead to the progressive transformation of normal cells into highly malignant cells. In this case, six major alterations are required for cancer progression: self-sufficiency in growth signals, insensitivity to growth-inhibitory (antigrowth) signals, evasion of programmed cell death (apoptosis), limitless replicative potential, sustained angiogenesis, and tissue invasion and metastasis [22]. In this context, it has recently been demonstrated that the activation of specific oncogenes concomitant with the loss of tumor suppressors in cortical astrocytes trigger cancer initiation with histological features similar to GBM [12]. As an example, the loss of INK4A/Arf associated with the activation of K-Ras and Akt in mature astrocytes lead to the formation of tumors closely related to GBM morphology. In this model, loss of INK4A/Arf induces the dedifferentiation of astrocytes which consequently become more sensitive to malignant transformation via activated oncogenes such as K-Ras [23]. At the same moment, another study strengthened the idea that astrocytes might be at the origin of malignant astrocytomas. Indeed, the combined loss of tumor suppressors p16(INK4a) and p19(ARF) enabled astrocytes to dedifferentiate in response to EGFR activation. Transduction of Ink4a/Arf(−/−) astrocytes with constitutively active EGFR induced a common high-grade glioma phenotype after xenografts experiments [24]. Last but not least, a very recent study demonstrated that the loss of p53 combined with oncogenes overexpression such as myr-Akt and c-Myc in mature astrocytes were highly tumorigenic in an *in vivo* model of brain tumor transplantation [25]. Loss of tumor suppressors and overexpression of oncogenes are not, stricto sensu, only involved in tumorigenicity since p53 and Akt have also been shown to induce the expression of stemness markers in mature astrocytes [26,27].

Loss of tumor suppressors and activation of oncogenes seem to be two mandatory criteria that both have to be met in order to trigger GBM initiation starting from astrocytes. Indeed, the only activation of oncogenes such as Ras and Akt is sufficient to induce GBM formation in nestine-positive progenitor cells but not in mature astrocytes [28]. In parallel, low grade gliomas can develop as a result of the inhibition of tumor suppressor Ink4a/Arf in nestine-positive progenitor cells but not in GFAP positive progenitors [29].

### 2.2. The Stem Cells Theory

Rudolf Virchow has described this second theory for the first time in 1863. Based on histological similarities between embryonic stem cells and cancer cells, Virchow proposed that tumors originally develop from “dormant” or quiescent cells located in the host tissue. From then on, the existence of such a fraction of cells has been described in many types of cancers [9] including brain tumors [10,11,12]. As a matter of fact, astrocytic gliomas contain a sub-population of cells which exhibits stem cell-like properties such as multipotentiality, the ability to self-renew or to form neurospheres *in vitro* [30,31,32]. Interestingly, growth properties of glioma-derived neurospheres *in vitro* were found to be significant predictors of tumor progression and clinical outcome [33].

In the same line, several genetic studies using murine glioma models and imaging analyses from clinical studies provided the evidence that GBM may arise from the SVZ stem cell niche (SVZ) [34,35,36]. This region notably maintains the ability to produce neurons and glia throughout life, functioning as a source of stem cells and progenitors in adults [17,18]. At this level, NSCs are hierarchically organized. Quiescent type B cells give rise to highly proliferative cells, also known as transit-amplifying progenitor cells (type C cells), which then differentiate into two lineage-restricted progenitor cells; neuroblasts (type A cells) and oligodendrocyte precursor cells (OPCs) [37,38]. In this context, tumor-initiating cells are thought to arise from quiescent type B cells located in the SVZ. Indeed, those cells were demonstrated to pile up the largest number of genetic mutations in a transgenic hGFAP-Cre/p53^flox^^/flox^ mouse model. Conversely, this study also showed that transit amplifying type C cells were able to accumulate strings of alterations which finally lead to tumor initiation and that Olig2-positive type C cells were notably involved in the early stages of gliomagenesis [39]. Additionally, another study recently showed that intraventricular infusion of PDGF was able to induce PDGFR alpha-positive type B cells to proliferate, contributing in this way to the generation of large hyperplasias exhibiting some GBM features [40]. In parallel, various studies have demonstrated the presence of human cytomegalovirus (HCMV) in GBM. This virus is now accepted as a tumor promoter in malignant brain tumor [41]. It has also been shown that HCMV preferentially infects NSCs. In this context, it has been hypothesized that NSCs’ modulation by HCMV may contribute to the brain tumor genesis [42]. However, there are no reports so far on how HCMV modulates the pre-tumorigenic environment of the brain.

Although the SVZ is usually considered to be the stem cell compartment for glioma formation in mice following the introduction of genetic alterations observed in adult malignant brain tumors [34,39,43], several other germinal zones in the brain could potentially be at the origin of brain tumorigenesis as well, including the third and the fourth ventricle [44,45]. For instance, it has been shown that pediatric gliomas are more likely to arise from NSCs located in the third ventricle [46]. This observation notably allowed us to shed the light on the crucial role of innate brain region NSCs’ heterogeneity in the patterning of gliomagenesis both in children and adults.

In 2009, the first example of a donor-derived brain tumor was reported. A boy with ataxia telangiectasia was treated with intracerebellar injection of human NSCs and was then diagnosed with a multifocal brain tumor four years after the treatment. Molecular and cytogenetic studies revealed that the tumor was derived from at least two donors, suggesting in this case the implication of NSCs in gliomagenesis [47]. This work was the first report of a human brain tumor complicating neural stem cell therapy but has also been nuanced by other studies which, nevertheless, do not minimize the role of stem-cell-like astrocytes during reactive neurogenesis after brain injury or disease and during brain tumorigenesis [48].

Finally, a very important study recently underlined the crucial role of NSCs in brain tumors and the relevance of initial genetic mutations in the pathogenesis. While recombination of PTEN/p53 in NSCs gave rise to gliomas, the deletion of either Rb/p53 or Rb/p53/PTEN generated primitive neuroectodermal tumors (PNET), indicating the significant role of the initial Rb loss in driving the PNET phenotype [43]. Futhermore p53, Rb and RTK were shown to be core-signaling pathways commonly activated in GBM [49].

### 2.3. The Midway Theory

However, despite the plethora of examples showing that both astrocytes and NSCs seem to be strong contenders involved in malignant brain tumor formation, the GBM cell of origin remains largely elusive. Recent studies have indeed shown that other non-stem cells, including NG2+ cells [50] and oligodendrocyte precursors (OPCs) [51,52,53], can also be viewed as potential cells for the origin of malignant glioma [54]. As a matter of fact, OPCs are the most dividing cells in the adult brain. Whether this means those progenitors are more susceptible to tumorigenicity enhancement is yet to be determined. However, their proliferative ability and their broad distribution in the white matter as well as the grey matter make those cells potential suspects in gliomagenesis. Favorable indications supporting this hypothesis can be found in the literature. OPCs are plastic cells that can be converted *in vitro* to immature multipotent cells able to give rise to neurons, astrocytes and oligodendrocytes [55]. Glioblastomas freely express NG2 and PDGFR, two markers closely associated with OPCs [56]. Moreover, PDGFRα signaling pathway, controlling proliferation and migration of OPCs, is commonly altered in GBM [7,49,57].

Very recently, mosaic analysis with double markers confirmed that malignant transformation generating GBM only occurred in OPCs in a mouse model where NSCs are homozygously mutated for p53 and NF1 [51]. Interestingly, the authors also reported for the first time that the GBM “cell of origin” could be distinct from the cell of mutation. It is therefore of major interest to find reliable candidate in order to promote quiescence and differentiation of OPCs. As a first recent example, treatment of primary murine GBM cells with agonists of Grp17 resulted in a decreased number of neurospheres [58]. Grp17 is a 7TM receptor involved in the differentiation of OPCs which can be activated by two classes of molecules such as uracil-nucleotides and cysteinyl-leukotrienes [59].

Following this controversy, it is thus of great importance to gather major attention on the GIC population in order to better understand their biology and origin(s) (Figure 1) to improve or develop new groundbreaking therapeutic strategies. The induction of GICs’ differentiation into less proliferative cells [60] along with the inhibition of signaling pathways involved in GICs’ proliferation [61] or even the disruption of the GICs’ relationship with their micro-environment [19] are as many hints which are given for further investigations.

## 3. The Tumor Micro-Environment

**Figure 2:**
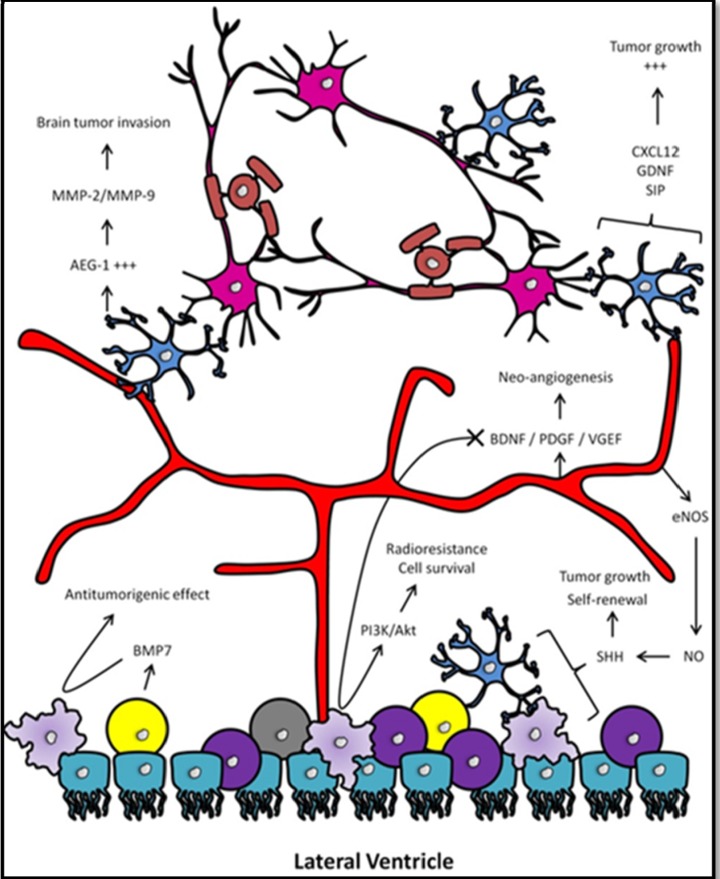
The tumor (SVZ) micro-environment. GICs (light purple) are regulated by the activation of oncogenes or by environmental conditions such as hypoxia or acidosis. GICs are able to express pro-angiogenic factors, such as VEGF (vascular endothelial growth factor), able to stimulate the outgrowth of new blood vessels (red) which will in the end help to maintain tumorigenicity. Endothelial cells are also able to produce nitric oxide in the endothelium through the activation of eNOS. Activated Notch signaling by nitric oxide in GICs is notably known to accelerate tumor progression. Defects in growth factor signaling pathways usually result in the activation of downstream growth factor signaling such as the PI3K/Akt signaling pathway which has been shown to be an important regulator of glioma cell survival and GICs’ radioresistant abilities. Neural progenitor cells (yellow) display antitumor effects by secreting tumor suppressors in the niche such as bone morphogenetic protein-7 (BMP7), inducing GICs’ differentiation. Astrocytes (dark blue) are closely associated with the vascular endothelium of the niche and were shown to secrete neurotrophic factors capable of driving the invasive properties of GBM cells (CXCL12, SIP, and GDNF). Astrocytes also express high levels AEG-1. Reports suggest that AEG-1 impacts on brain tumor invasion notably through the activation of MMP-2 and MMP-9.

Glioblastomas are made of heterogeneous cell populations which do not only catch external signals from the environment but also respond to the latter in order to take advantage of it. It is commonly accepted that tumor-associated parenchymal cells such as vascular cells, microglia, peripheral immune cells and neural precursor cells directly interact with GBM cells and play a crucial role in controlling the course of the pathology. In the following paragraphs, we will try to describe the multiple interactions between the GIC population and the parenchymal cells in order to highlight the pathological impact of the tumor micro-environment on malignant brain tumors (Figure 2).

### 3.1. Involvement of Microglia

Tumor-associated macrophages are the most predominant inflammatory cell type which infiltrate GBM [62] and account for the major non transformed cell population in GBM biopsies [63,64]. Tumor-associated microglia notably break into the tumor mass in response to chemo-attractive cytokines released by the tumor it-self such as monocyte chemotactic protein-3 (MCP-3), colony-stimulating factor 1 (CSF-1) and granulocyte-colony stimulatory factor (G-CSF) [65,66]. Malignant brain tumors take advantage of this situation since tumor-associated macrophages were shown to infiltrate the tumor in order to enhance GBM cells’ invasion by degrading the extracellular matrix. Indeed, microglia is able to trigger the release of membrane Type 1 metalloprotease (MT1-MMP) in response to soluble factors secreted by GBM cells which, in turn, release matrix metalloprotease 2 (MMP-2) that will be fully activated by the microglia MT1-MMP [67]. Matrix metalloprotease 2 is notably upregulated in microglia following the activation of the CX3CL1/CX3CR1 signaling pathway. Interestingly, chemokine receptor CX3CR1 was also shown to be upregulated in glioma associated microglia [68]. Moreover, a recent study showed that the common CX3CR1 allelic variant, termed V249I, was associated with increased GBM survival and reduced microglial cell infiltration in primary tumor biopsies as well [69]. All these findings definitely demonstrated the importance of microglia in GBM invasive properties and the necessity for developing more reliable *in vivo* models. We are convinced that better *in vivo* models would definitely improve our knowledge on those invasive tumor (initiating) cells which escape neurosurgery and radiotherapy modalities by leaving the tumor bulk.

### 3.2. Involvement of the Immune System

Following the example of tumor-associated macrophages, lymphocytes were also reported to infiltrate human gliomas. It has recently been suggested that a specific subtype of lymphocytes, regulatory T cells or Tregs, play an important role in the regulation of the immune response. In 2007, El Andaloussi and Lesniak described a positive correlation between the progression of the disease and the presence of Tregs in tumors with high malignancy [70]. Once again, chemokines such as CCL2 and CCL22 were incriminated for the attraction of Tregs towards the tumor site [71]. This specific infiltration was correlated with an increase in TGF-β1 mRNA and protein expression in a model of intracranial xenografts. The crucial role of the brain environment was markedly put to light in this study since this correlation was not found in gliomas injected subcutaneously [72]. For those reasons, Tregs-associated glioma have been the center of attention for decades and represent nowadays innovative target in glioma therapy.

### 3.3. Involvement of Neural Progenitors

Throughout the last decade evidence is accumulating that glioblastomas also interact with neural progenitor cells (NPCs) in the micro-environment. It has indeed been widely demonstrated that endogenous NPCs, from the subventricular zone or from the *corpus callosum*, preferentially home to experimentally induced brain tumors [73,74,75,76] probably in a CXCR4 dependent-manner [77]. Those tumor-associated NPCs are in fact diverted from their physiological migratory path in order to end up their journey in cellular layers surrounding the tumor mass [76]. There, they display important antitumorigenic effects by releasing soluble factors which interfere with GBM cell proliferation [78,79], causing GBM cell death [76] and promoting GICs’ differentiation [73]. Moreover, significant survival improvements were observed *in vivo* using orthotopic coinjection of NPCs together with glioblastoma cells. This study also demonstrated that the tumor-suppressor effect of NPCs is largely related to aging and neurogenic abilities since younger mice significantly outlived older ones. Strikingly, this survival default was sealed by inoculating GBM cells along with NPCs in older mice brains suggesting the close relationship between NPCs’ antitumorigenic properties and neurogenic aptitudes [76]. Let’s keep in mind that the antitumorigenic capacity of NPCs has only been described in rodent models. It seems therefore required to check if human NPCs also display antitumorigenic properties similar to what has been described in rodents so far, especially since aging is considered as one of the most important prognostic factor for the disease. Moreover, the fact that neurogenesis declines with aging in humans [80] strengthens the link between GBM and this prognostic factor even more, notably by potentially decreasing the amount of NPCs and their related antitumorigenic effects throughout lifespan.

### 3.4. Involvement of the Vascular Niche

In physiological context, NSCs are located in specific regions of the brain called neurogenic niches [81,82]. Those niches, usually defined by a large vasculature network, have been the center of attention for many years since these anatomical structures were demonstrated to be the stem cell niches for normal and malignant neural tissue as well. Indeed, just like the adult NSCs, GICs also rely on vascular niches in order to control the balance between self renewal capacities and differentiation [19,83,84]. Moreover, let’s just not forget that high grades glioma are mainly characterized by hallmarks such as endothelial hyperplasia and microvascular proliferation which are associated with a transition to a more aggressive phenotype, making malignant gliomas among the most vascularized tumors [85].

The vascular niches have been shown to be the primary location for cancer cells with stem cell-like characteristics [86]. In parallel, it has recently been shown that GICs can acquire a specific endothelial phenotype in order to create an early bound between the vasculature network and the tumor mass [87]. Moreover, GICs preferentially associate with endothelial cells which, in turn, accelerate their tumorigenic capacities. In fact, endothelial cells were shown to selectively interact with the GIC population in culture and supply them with secreted factors which maintain these cells in a self-renewing and undifferentiated state. Moreover, increasing the number of endothelial cells or blood vessels in orthotopic brain tumor xenografts expanded the pool of self-renewing cancer stem cells and accelerated the initiation and growth of tumors [19]. Interestingly, protein ligands that are found within the vascular niche have been demonstrated to regulate both stem cell self-renewal and angiogenesis, putting forward the idea that these two processes are related. For instance, KIT ligand, also known to be a stem cell factor, was shown to be a powerful GBM-derived proangiogenic factor also involved in migration, survival and proliferation of NPCs [88,89]. In parallel, pigment epithelium-derived factor (PEDF) has also been demonstrated to play a crucial role in angiogenesis and to be involved in NSC self-renewal [90,91]. More recently, SVZ blood vessels and the ependymal cell layer of the vascular niche were shown to secrete CXCL12, creating in this way a u-shaped gradient in the niche [92]. Besides, chemokine receptor CXCR4 is known to be preferentially expressed by GICs [93] as well as the entire SVZ lineage [92]. In this study, the authors notably speculated that high levels of CXCL12 in the ependymal layer could help to promote quiescence. Indeed, high levels of CXCL12 were shown to result in receptor internalization, desensitization, and quiescence of hematopoietic stem cells, whereas lower concentrations resulted in proliferation and differentiation [94].

Following these observations, it has been suggested that the molecular crosstalk between GICs and the vascular network of the niches plays a critical role in tumor progression. A better understanding of these lines of communication will definitely provide new insights to improve the actual therapeutic means and develop new therapies which better target the micro-environment.

As a clinical example, there are considerable paracrine interactions between endothelial cells and the brain tumor cells in the micro-environment notably through the release of endothelial-derived soluble factors such as VEGF [95]. This factor has been shown to mediate the intercellular crosstalk between GICs and the tumor endothelium in order to induce angiogenesis [96]. Interestingly, neo-angiogenesis in astrocytomas reflects the tumor grade and is therefore often correlated with the poor prognosis or the aggressive phenotype of the disease. Furthermore, the increased amount of VEGF in the tumor micro-environment has been demonstrated to enhance the ability of GICs to promote angiogenesis compared to the non tumor-initiating cell populations [96,97]. Although the molecular mechanisms underlying this increase of VEGF production remain unclear, environmental factors such as hypoxia or acidosis have been proposed to play an important role in this process [98,99]. Activation of oncogenes such as EGFR or loss of PTEN can also lead to higher levels of VEGF in malignant gliomas [100]. As a result, the use of bevacizumab, an anti-VEGF antibody, allowed to significantly reduce tumor angiogenesis both *in vitro* and *in vivo* [101]. It is possible that this drug directly disrupts the maintenance of GICs and, in this way, effectively eliminates the roots of tumor progression. Although data on randomized phase III clinical trials with anti-angiogenic molecules are not yet available, this treatment regimen is already applied in several clinical centers at the time of recurrence (NCT00671970 and NCT00350727, [102]). Our opinion is that future anti-angiogenic therapies will have to rely on strategies combining chemotherapy and drugs which target invasive GBM cells. Indeed, those cells, sometimes located far away from the highly vascularized tumor core, are notably not targeted by anti-angiogenic therapies.

## 4. The Human SVZ and Its Clinical Implications in GBM

**Figure 3:**
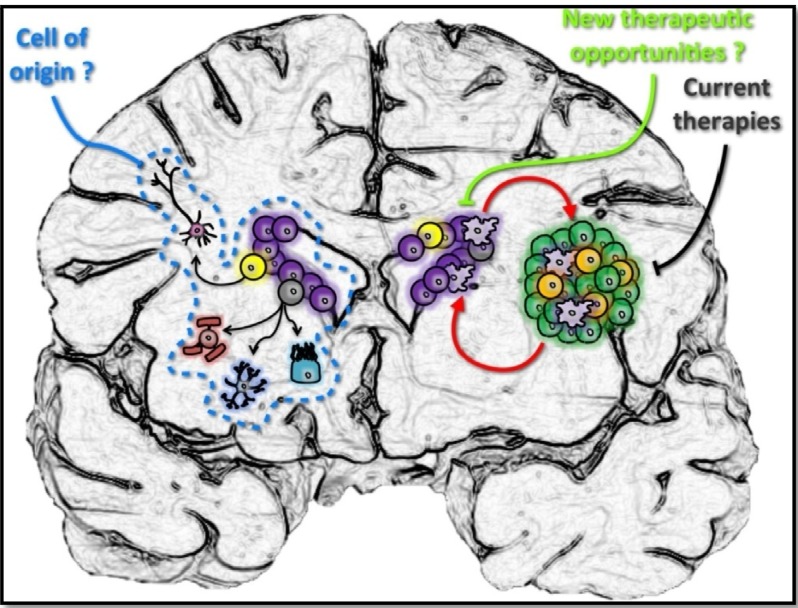
Relationship between the adult neurogenic niches and glioblastoma-initiating cells. GICs (light purple) have been found to play an important role in GBM aggressiveness and the resistance of the tumor to current treatments, making them an attractive target for therapeutic interventions. However, the design of new GIC inhibitors has proven difficult because their definition is nowadays still confused by the twilight zone of their origin. Indeed, whether GICs are derived from NPCs (yellow) or differentiated/mature cells remains uncertain. In any case, the vascular niche offered by the brain SVZ might be of interest to maintain GICs’ features, even if they are not indigenous. Undoubtedly, particular characteristics of this specific niche and its localization away from the tumor mass make it a significant resource for tumor recurrence. It is expected that future therapies targeting GBM, GICs and their specific environment will help to increase the dramatic survival rate of GBM patients.

The discovery by Eriksson *et al*., in 1998, of neural progenitor cells (NPCs) capable of becoming mature neurons in the human brain, thought for decades to be a quiescent organ, has brought the brain’s plasticity into sharp focus [103]. However, researches about stem cells implication in neurological disorder repair have met little success so far and their capacity to regenerate neurons after a lesion is, for now on, very limited. Indeed, NPCs were only found to replace interneurons in specific regions of the brain such as the olfactory bulbs or the *dentatus gyrus*. Human NPCs, which look like glial cells but with stem cell features, remain in the adult brain in two restricted regions after that the hippocampal sulcus has become the subgranular zone (SGZ) of the hippocampus and the lateral ganglionic eminences turned into the SVZ [104,105]. Because neurogenesis in the SGZ is rigidly fixed by the age of 30 and is composed of a very small number of cells and nor could a link be established between the hippocampus and brain tumors, we will only focus on the SVZ environment in this review. As a matter of fact, the SVZ is the region that borders the ependymal layer on the lateral wall of the lateral ventricle and is separated from the caudate nucleus by a layer of myelin [106]. In the late 90s, specific culture conditions, using neurosphere formation, were used in order to isolate cells from the lateral wall of the temporal lobe in epileptic patients. These experiments already suggested at that time the presence of human NSCs in the adult brain [107,108,109]. As shown in rodent, progenitor cells located in this specific area are able to produce neuroblasts which migrate and integrate the olfactory bulbs. However, it seems that there is a considerably less activity in the human SVZ compared to rodents. Nevertheless, those human progenitors have the ability to proliferate and migrate towards injured regions close to the SVZ. This should be taken into consideration for the development of new treatment in neurological disorders and for our basic understanding of GBM (Figure 3).

The adult human SVZ hosts three types of cell harboring progenitor properties (A, B and C) as already mentioned in the previous section [110]. Type C cells are found in the deepest layer (regarding the ventricle’s wall) close to the myelin compartment. Type B cells are located in a well-defined cell-rich region. Type A cells staid in the cell-poor layer immediately beneath the ependymal layer. The ratio of cell types between rodent and human differs with the particularity that type A cells, or the migrating neuroblasts, are the most abundant in rodents while type B cells, identified as the most quiescent primary progenitors in rodents, are the major type in human [111,112]. Type C cells were shown to be less numerous in both species.

In rodents, the migration of type A cells to the olfactory bulb to replace interneurons is well established [113,114,115]. Recently, Curtis and collaborators have extended this knowledge to humans and discovered that human neuroblasts are also able to leave the SVZ and reach the olfactory bulbs trough the rostral migratory stream (RMS), a vestigial lumen that connects the lateral ventricle to the olfactory bulb [116,117]. The human RMS harbor neurogenic properties with a large number of cells proliferating found on the road to the olfactory bulbs [118,119].

To date, many studies in rodents have currently supported the idea that the “cell of originin” malignant brain tumors could derive from SVZ progenitors. Unfortunately the biology and the precise contribution of neural progenitors to normal human brain functions remain to be addressed and the understanding of their roles in neurological diseases has just started. Beyond the hypothetic role of the SVZ in generating GBM, it could be that GICs do not originate from NPCs. Using bio-mathematical models, Bohnam and collaborators discovered that 50% of GBM are actually located away from the SVZ environment and that their SVZ origin would therefore be doubtful [120]. No matter what this study shows, let’s not forget that the SVZ offers a specific environment for GICs, as described in the previous section, which could directly or indirectly be involved in GBM growth and help to escape conventional treatment which finally account for tumor recurrence. Moreover, evidence accumulating from clinical observations push in that direction as well and tend to evidence bit by bit the relationship between the SVZ and malignant brain tumors (Figure 3).

In 2007, Lim and collaborators reported for the first time the association between lesions surrounding neurogenic niches and the tumor phenotype [35]. Based on GBM MRI classification, they showed that tumors connecting the SVZ (almost 50%) are more frequently characterized as multi-focal tumors. At recurrence, tumors connecting neurogenic niches also favor new lesions at greater distance. It is of course tempting to speculate that those SVZ contacting tumors derived from NPCs. However there is no evidence so far that SVZ-related tumors are more likely to derive from stem cells, using for instance GBM genetic analysis [121]. In contrast, immune system genes were significantly over-expressed in tumor connected to the SVZ. This study by Kappadakunnel and collaborators failed to confirme that tumors which reach the SVZ display a more invasive phenotype at recurrence. The small size of the cohort was proposed as an explanation for this lack of reproducibility.

The SVZ involvement has been assessed as a potential independent prognostic factor for the overall survival (OS) and the progression free survival (PFS) in GBM patients [121,122,123,124]. Radiological observations of an intimate contact with the SVZ demonstrated an association with poor survival rates. An interesting study reported a significant decrease of survival in patients bearing tumors connected to the lateral ventricle [122]. However, the observed median survival difference (8 *vs.* 11 months) did not show statistical signification after that patients had undergone surgery (11 *vs*. 14 months). In a same line, SVZ connexion profile, analyzed in a cohort of 47 GBM patients, failed to demonstrate a significant correlation with survival [121]. However, a trend to a shorter survival rate was once again observed when GBM cells invaded the SVZ environment (median OS of 358 *vs.* 644 months). Recently, Kaplan-Meier analyses on a cohort of 91 GBM patients demonstrated shorter PFS at 6 months (47% *vs.* 69% survivors) as well as shorter OS at 2 years (23% *vs.* 48% survivors) in the group of patients whose tumors were connected to the SVZ compared with patients harboring no SVZ lesions [124]. This study also explored the impact of a cortical involvement and reported that such a relation does not exist. Nevertheless, conclusions reached by independent studies differ widely and the precise meaning of this phenotype is not clear yet. Cohort homogeneity may have contributed to this controversy. Tumor sizes were shown to be different between tumors classified according to the SVZ contact [125] but tumor volume did not impact survival [126]. However, the type of surgery performed, temolozomide adjuvant chemotherapy protocol and Karnofsky performance status score (KPS) are well-established independent prognostic factors of the disease [127,128,129]. Be that as it may, but the human SVZ has to be taken into consideration speaking about GBM.

Radiotherapeutic data also support the claim that GBM are related to the SVZ. A group from the University of California Los Angeles (UCLA) tested the hypothesis that targeting adult neurogenic niches could be of benefit for GBM patients in a retrospective study of 55 patients, including 17 patients with grade III and 38 patients with grade IV histology [130]. Aside the small size of the study and the lack of crucial information, the authors reported that a >43Gy irradiation dose of the ipsi- and contralateral SVZ increased the median progression free survival (15.0 *vs.* 7.2 months). Another retrospective study measured the SVZ dose-volume parameters and found a correlation with the survival outcome of 40 patients with GBM [131]. Multivariate Cox regression analyses for important prognostic factors (age, KPS, surgery type) revealed that higher ipsilateral SVZ irradiation doses were not found to be independent predictors for PFS but for OS. Furthermore, higher irradiation of the contralateral SVZ (>57.9 Gy) was associated with worse prognosis. Likewise, it has also been shown by another study that the SVZ involvement during radiotherapy is an independent predictive factor for shorter PFS and OS [123]. More recently, the UCLA group confirmed their previous data about the impact of SVZ irradiations on PFS and OS on a larger cohort of 173 patients by Cox regression analyses including five covariates (ipsilateral and contralateral SVZ doses, clinical target dose, age and extent of resection) [132]. Again, a significant correlation was found between high ipsilateral SVZ irradiation and improved survival, both for PFS and OS. Multivariate analysis only confirmed this advantage for PFS. Last but not least, a recent retrospective study confirmed these trends and specified that patients with GBM are more likely to benefit from SVZ irradiation when gross total resection was performed [133]. In this case, PFS was significantly higher in patients receiving ipsilateral SVZ doses of 40 Gy or above (15.1 *vs.* 10.3 months). Interestingly, OS was also significantly improved in patients receiving ipsilateral SVZ doses of 40 Gy or above (17.5 *vs.* 15.6 months). Such benefits could not be observed in patients treated with biopsy and subtotal surgery. Obviously, those retrospective studies do not seem to be sufficiently robust to provide assurance that there is a consistent role of the SVZ contact in the GBM tumorigenicity.

## 5. Conclusions

While it is apparent that targeting GICs and neurogenic niches, given their particular architectures, should be seen as a great opportunity to improve the survival of GBM patients, critical data are nowadays still lacking. Indeed, further studies inquiring the origin(s) and the exact definition of GICs as well as robust prospective clinical trials are mandatory. We are convinced that a better understanding of the relationship between GICs and the so called neurogenic niches will provide new insights in order to improve or to set up new therapeutic strategies for highly malignant brain tumors. Indeed, even if the percentage of patients who survive two years from diagnosis of GBM has more than tripled in the last five years, largely because of the use of temozolomide plus radiation in addition to progress made with bevacizumab, research on treatment options for GBM is way more exciting now than ever before. Nowadays, the development of state-of-the-art neuroimaging techniques for improved surgical resections, vaccines and therapies aiming molecular targets as well as signaling pathways are bit by bit bending the tail end of the curve and raise great hope of making major improvements for GBM patients.
